# Advanced Therapies for the Management of Immune Checkpoint Inhibitor-Induced Colitis

**DOI:** 10.3390/cancers18193108

**Published:** 2026-09-24

**Authors:** Cosimo Simone Matichecchia, Fabrizio Fanizzi, Alessandra Zilli, Roberto Ferrara, Sara Oresti, Luca Albarello, Mariangela Allocca, Laurent Peyrin-Biroulet, Silvio Danese, Ferdinando D’Amico

**Affiliations:** 1 Department of Gastroenterology and Endoscopy, IRCCS Ospedale San Raffaele, 20132 Milan, Italy; matichecchia.cosimos@hsr.it (C.S.M.); fanizzi.fabrizio@hsr.it (F.F.); zilli.alessandra@hsr.it (A.Z.); allocca.mariangela@hsr.it (M.A.); danese.silvio@hsr.it (S.D.); 2 Faculty of Medicine and Surgery, Vita e Salute San Raffaele University, 20132 Milan, Italy; 3 Department of Medical Oncology, IRCCS Ospedale San Raffaele, Università Vita-Salute San Raffaele, 20132 Milan, Italy; ferrara.roberto@hsr.it (R.F.); oresti.sara@hsr.it (S.O.); 4 Pathology Unit, IRCCS Ospedale San Raffaele, 20132 Milan, Italy; albarello.luca@hsr.it; 5 Northwestern Medicine, Digestive Health Institute, Chicago, IL 60611, USA; peyrinbiroulet@gmail.com

**Keywords:** ICI-colitis, advanced therapy, infliximab, vedolizumab, IBD, biologics

## Abstract

Immune checkpoint inhibitors (ICIs), including anti-CTLA-4, anti-PD-1, and anti-PD-L1 agents, have revolutionized cancer therapy by enhancing T-cell–mediated antitumor responses and significantly improving survival across multiple malignancies. Despite these advances, ICIs are associated with immune-related adverse events (irAEs), with ICI-induced colitis representing one of the most common and severe gastrointestinal toxicities. Clinically and histopathologically, this condition often resembles inflammatory bowel disease (IBD) and can substantially impact treatment continuity and patient quality of life. Corticosteroids remain the standard first-line therapy; however, up to one-third of patients exhibit steroid-refractory disease or develop significant steroid-related toxicity, highlighting the need for alternative therapeutic strategies. Biologic agents, particularly infliximab and vedolizumab, have demonstrated high efficacy in this setting, with infliximab associated with more rapid symptom control and vedolizumab offering a favorable steroid-sparing profile. In patients with multi-refractory disease, additional agents such as ustekinumab, tofacitinib, and tocilizumab have shown promise, although current evidence is limited and primarily derived from small studies and case series. This review critically examines current treatment paradigms for ICI-induced colitis, focusing on efficacy, safety, and potential implications for oncologic outcomes. Despite increasing use of advanced therapies, clinical decision-making remains largely guided by retrospective data, and key uncertainties persist regarding optimal treatment sequencing, patient selection, and long-term safety. Future research should prioritize prospective randomized trials incorporating both oncologic and gastrointestinal endpoints, as well as mechanistic studies aimed at identifying predictive biomarkers and therapeutic targets that preserve anti-tumor immunity.

## Simple Summary

Immune checkpoint inhibitors help the body’s immune system attack tumors and have greatly improved survival for many cancer patients. However, by boosting the immune system, these drugs can also cause it to attack healthy tissue, and inflammation of the bowel is one of the most common and serious side effects. Steroids are usually given first, but they do not work for many patients or can cause their own problems with long-term use. Other drugs, originally developed for bowel diseases such as Crohn’s disease and ulcerative colitis, are increasingly used instead, but gaps of evidence persist on which drug to choose, in what order, and whether these treatments might weaken the cancer drug’s effect.

## 1. Introduction

Immune checkpoint inhibitors (ICI), such as anti-cytotoxic T-lymphocyte-associated antigen-4 (CTLA-4) antibodies, anti-Programmed Death-1 (PD-1) agents, and anti-Programmed Death-Ligand 1 (PD-L1) drugs have revolutionized cancer treatment [1,2,3]. These therapies block inhibitory signals from T cells, thereby enhancing their activation, proliferation, and cytotoxic function to elicit robust, durable anti-tumor immune responses [1,4,5]. Initially approved for advanced melanoma, they are now approved or under investigation for numerous indications including non-small cell lung cancer, hepatocellular carcinoma, gastric and esophageal cancers, and mismatch repair-deficient colorectal tumors [1,6,7].

Moreover, these therapies have significantly improved overall survival rates, have achieved long-term remissions in subsets of patients with metastatic disease, and increasingly serve as first-line or adjuvant standards, often supplanting traditional chemotherapy [1,8,9]. The most pronounced survival gains, however, now appear in the perioperative (neoadjuvant and adjuvant) setting, where treatment is delivered with curative intent [10]. In resectable non-small cell lung cancer, neoadjuvant nivolumab plus chemotherapy raised the 5-year overall survival (OS) rate to 65.4% versus 55.0% with chemotherapy alone (HR 0.74) [11]. Similarly, in the same setting, a perioperative pembrolizumab regimen conferred a significant survival benefit: the 5-year OS rate was 64.6% versus 53.6% (HR 0.74) [12]. Beyond lung cancer, comparable advantages have emerged in resectable stage III melanoma, where a neoadjuvant combination of ipilimumab + nivolumab improved event-free survival (83.7%) compared to adjuvant therapy alone (57.2%) after 12 months [13]. Likewise, in resected esophageal or gastroesophageal junction cancer, adjuvant nivolumab prolonged disease-free survival following neoadjuvant chemoradiotherapy [14].

Despite their efficacy, this immune unleashing often results in immune-related adverse events (irAEs) such as cutaneous rash and pruritus, endocrinopathies (e.g., hypothyroidism), pneumonitis, and hepatitis, with ICI-induced colitis emerging as one of the most prevalent and severe gastrointestinal toxicities, frequently resembling inflammatory bowel disease (IBD) clinically, endoscopically, and histopathologically [15,16,17].

The incidence of ICI-induced colitis varies markedly depending on the therapeutic regimen employed, ranging from 0.7% to 1.6% with anti–PD-1 agents and 5.7% to 9.1% with anti–CTLA-4 agents and reaching 13.6% when both were used in combination [3], sometimes necessitating treatment discontinuation [18].

ICI-induced colitis is primarily managed with corticosteroids as first-line therapy; however, long-term exposure to corticosteroids results in different side effects [17]. In addition, a significant proportion of patients with ICI-induced colitis fail to respond adequately to first-line corticosteroid therapy, with estimates ranging from approximately 21% to as many as two-thirds of patients [19,20]; for these reasons, steroid-sparing strategies for the treatment of ICI-induced colitis, which include the use of biologic agents and JAK inhibitors, are increasingly used [5,18].

Controversies remain on optimal timing of advanced therapies versus steroids and potential impacts on anti-tumor responses [17,21].

This review aims to critically assess current treatment paradigms for ICI-induced colitis, evaluating efficacy, safety, and cancer implications, focusing on available advanced therapies.

## 2. Materials and Methods

This work was conceived as a narrative, expert-led review; no protocol was registered, and no formal risk-of-bias assessment or quantitative synthesis was undertaken. The aim was to provide an interpretative synthesis of the available evidence on advanced therapies for ICI-induced colitis, integrating clinical, mechanistic, and guideline-level sources, and to situate that evidence within contemporary oncological practice. A structured literature search was performed in PubMed/MEDLINE, Embase, and Scopus from database inception to 30 June 2026. The full search strategy, including database-specific MeSH and Emtree terms, Boolean operators, and eligibility criteria, is reported in Supplementary Table S1. In brief, terms relating to immune checkpoint inhibitor-induced colitis and enterocolitis were combined with terms relating to inflammatory bowel disease and to advanced therapies, including biologic agents and small molecules. Only articles published in English were considered. Original studies, systematic reviews, meta-analyses, consensus statements, and clinical practice guidelines were eligible; narrative articles were consulted for context but were not used as the primary source for quantitative statements. Publications not pertinent to the topic or reporting insufficient methodological detail to permit interpretation were excluded. Titles and abstracts were screened independently by two authors (C.S.M. and F.F.), followed by full-text assessment of potentially relevant records; inter-rater agreement was not formally quantified, consistent with the narrative design of this review. Reference lists of retrieved articles and of relevant reviews were hand-searched to identify additional pertinent studies.

## 3. Results

### 3.1. Pathogenesis

Immune checkpoint inhibitor-induced colitis results from dysregulated immune activation following blockade of CTLA-4 and PD-1/PD-L1 pathways, which removes inhibitory signals and leads to increased cytotoxic T-cell activity against colonic antigens [3,15]. The condition is driven by the expansion and activation of interferon-gamma-producing CD8+ tissue-resident memory T cells and polyfunctional CD4+ T cells, causing mucosal inflammation and epithelial barrier dysfunction [16,17]. The gut microbiota plays a vital role, as microbiota-dependent activation of CD4+ T cells and depletion of regulatory T cells are central to the development of ICI-induced colitis [18]. The IL-23/IFNγ axis is a key pathway, with blockade of this axis reducing colitis in preclinical models [19]. Epithelial cell apoptosis, increased cell turnover, and immune-epithelial crosstalk contribute to barrier dysfunction and malabsorption [17,20]. Tight junction integrity is compromised through myosin light chain kinase 1 (MLCK1)-mediated pathways, with tumor necrosis factor (TNF) secreted by CD8+ and CD4+ T cells acting as upstream regulators [21].

### 3.2. Clinical Presentation and Diagnosis of ICI-Induced Colitis

ICI-induced colitis presents with diarrhea as the hallmark symptom, which may be accompanied by abdominal pain, cramping, urgency, blood and mucus in the stool, fever, nausea, vomiting, and loss of appetite [18,22,23]. The median time to onset is approximately 6 weeks after therapy start, and symptoms can escalate rapidly over days, particularly with anti-CTLA-4 agents like ipilimumab [22,23]. Severe cases can progress to life-threatening complications, including toxic megacolon, ileus, peritonitis, bowel perforation, and death, though mortality from gastrointestinal immune-related adverse events is rare [22]. Severity is graded using the CTCAE, which classifies toxicity from grade 1 (mild) to grade 5 (death) [22]. Grade 1 is defined as fewer than four bowel movements above baseline per day without colitis symptoms; grade 2 is defined as 4–6 bowel movements above baseline with colitis symptoms not interfering with activities of daily living; and grades 3–4 are defined as more than 6 bowel movements above baseline, interference with activities of daily living, hemodynamic instability, hospitalization, or serious complications [22]. However, symptoms typically correlate poorly with endoscopic severity, radiologic findings, and response to treatment, and the clinical utility of CTCAE grading for immune-related adverse events has not been fully established [24]. The diagnostic workup includes stool evaluation to exclude infectious etiologies (C. difficile, nucleic acid amplification tests for gastrointestinal pathogens, and ova and parasites in appropriate clinical contexts) [22,23]. Fecal calprotectin or lactoferrin testing can help stratify patients, with the sensitivity of stool lactoferrin reaching 90% for histologic inflammation [22]. Endoscopy with biopsy is the reference standard for diagnosis and should be considered before initiating high-dose systemic glucocorticoids, as endoscopic findings predict treatment response [22] (Figure 1); however, flexible sigmoidoscopy is often adequate since approximately 95% of patients have left-sided colonic inflammation [22]. Abdominal/pelvic CT with contrast should be considered in cases with high suspicion for complications such as toxic megacolon, abscess, or perforation [23]. Laboratory blood tests, including complete blood count, comprehensive metabolic panel, and inflammatory markers, may be performed, though they are rarely specific for ICI-induced colitis [22]. Importantly, ICIs can also cause reactivation of quiescent IBD in patients with pre-existing ulcerative colitis or Crohn’s disease, and this must be distinguished from de novo ICI-induced colitis [25]. A meta-analysis including 193 patients with pre-existing IBD treated with ICIs found that approximately 40% experienced IBD relapse, even among patients with documented clinical and endoscopic remission prior to ICI initiation [26]. Moreover, in a large multicenter retrospective study, gastrointestinal adverse events occurred in 41% of IBD patients compared to 11% in matched controls without IBD (*p* < 0.001), with a median time from last active IBD episode to immunotherapy initiation of 5 years [27]. Among patients who experienced IBD relapse, 76% required corticosteroids and 37% required biologic therapy, with gastrointestinal perforation occurring in fewer than 5% of cases [26]. Despite the increased risk of GI adverse events, preexisting IBD is not necessarily an absolute contraindication to potentially life-saving immunotherapy, with tumor responses appearing comparable to those in patients without IBD [22,28]. Histopathologically, ICI-induced colitis is distinguished from IBD by CD8+ T-cell–dominant infiltration (vs. CD4+ in IBD), with a CD8/CD4 ratio cutoff of 1.17 showing 83% sensitivity and 84% specificity [29]. ICI-induced colitis also demonstrates significantly less basal plasmacytosis (14% vs. 92%, *p* < 0.0001) and crypt distortion (23% vs. 75%, *p* = 0.003) compared to ulcerative colitis, but more prominent crypt epithelial apoptosis, reflecting its acute rather than chronic nature [28,30].

### 3.3. Management of ICI-Induced Colitis

The management of ICI-induced colitis follows a graded approach based on symptom severity to prevent rapid progression to life-threatening complications such as perforation or toxic megacolon [3,18]. Mild cases are managed supportively with antidiarrheal agents, fluid and electrolyte replacement, and continuation of ICI therapy, whereas moderate-to-severe cases require withholding ICI treatment and initiating systemic glucocorticoids, typically at doses of 0.5 to 1 mg/kg/day of prednisone or equivalent [3]. Prolonged corticosteroid therapy carries substantial risks of adverse events, including opportunistic infections, osteoporosis, hyperglycemia, proximal myopathy, psychiatric disturbances, and adrenal insufficiency [5,17]. Moreover, corticosteroids may blunt antitumor immune responses by suppressing T-cell activation and proliferation, potentially reducing ICI efficacy and impacting oncologic outcomes [17,21,31]. In a post hoc analysis of individual patient data from 834 patients treated with immunosuppression for treatment-related adverse events across six registrational phase II/III trials of combined anti-PD-1 and anti-CTLA-4 therapy, a higher corticosteroid peak dose was independently associated with worse progression-free and overall survival, whereas cumulative corticosteroid dose was not associated with survival [32]. A nationwide retrospective cohort study found that a high dose of prednisone at the start of tapering (≥75 mg/day) was associated with increased mortality (HR 1.67, 95% CI 1.04–2.69, *p* = 0.035) [33]. Consequently, there is growing advocacy for steroid-sparing strategies that employ biologics as first-line or early therapies to mitigate these risks while potentially preserving or enhancing ICI antitumor efficacy [21].

Biologic agents, particularly anti-tumor necrosis factor therapy with infliximab and integrin receptor blockade with vedolizumab, are currently recommended by major guidelines. They have emerged as the primary advanced therapeutic options for steroid-refractory or steroid-dependent ICI-induced colitis, offering targeted immunosuppression with distinct safety and oncologic profiles [5,21].

#### 3.3.1. Infliximab

Infliximab is recommended as second-line therapy for ICI-induced colitis when patients fail to respond to high-dose corticosteroids within 3–5 days (specifically, no response within 72 h or incomplete response within one week) or in cases with high-risk endoscopic features such as colonic ulceration [22,23,34]. The standard dosing regimen is 5 mg/kg intravenously at weeks 0, 2, and 6, following IBD protocols, with the full three-dose induction regimen recommended to maximize mucosal healing, prevent symptomatic relapse, and limit corticosteroid exposure, particularly in patients with deep ulceration [22,28]. While a single dose may resolve symptoms in some patients, others require a second dose due to incomplete response or relapse, and receiving more than three doses is associated with less frequent colitis recurrence compared to fewer doses [28]. Efficacy is substantial: a systematic review and meta-analysis conducted by Ibraheim et al. pooling 333 patients treated with infliximab across 17 studies reported an overall infliximab response rate of 81% (95% CI 73–87, *p* = 0.01), with response favorable both in patients treated with anti-PD-1/PDL-1 monotherapy (*n* = 6, 100%) and anti-CTLA-4-containing regimens (78%, 95% CI 68–85, *p* = 0.003) [35]. A retrospective cohort study including 140 cancer patients demonstrated complete remission rates of 73% after two or more doses, with median symptom improvement occurring within 3 days (IQR 2–4) and complete remission within 31 days (IQR 14–61), which is notably faster than the response seen in IBD [33]. Importantly, early administration of infliximab is associated with significantly shorter time to symptom resolution compared to corticosteroids alone: in a single-center retrospective study of patients with ICI-induced colitis, early addition of infliximab resulted in median time to diarrhea resolution of 3 days versus 9 days with corticosteroids alone, despite higher-grade colitis in the infliximab group [36]. As these findings derive from one retrospective cohort without randomization, their generalizability is limited. This steroid-sparing effect is clinically significant, as high corticosteroid doses (≥75 mg/day prednisone at taper initiation) are associated with increased mortality (HR 1.67, 95% CI 1.04–2.69) [33]. When infliximab is used, an attempt to taper steroids in less than 2 to 4 weeks should be made to minimize infection complications [22]. Safety considerations include a 24% rate of infection-related hospitalization and 16% incidence of secondary gastrointestinal infections, with Clostridioides difficile being the most common (64% of secondary infections) [33]. Additionally, 10% of patients experience thromboembolic events within 90 days of infliximab treatment [33], a rate likely driven by the inherent prothrombotic state conferred by active inflammation and the underlying tumor rather than representing a direct effect of the drug. Before initiating infliximab, infectious disease screening (HIV; hepatitis A, B, C) and tuberculosis testing (QuantiFERON-TB Gold or T-Spot) should be performed [22,28]. Infliximab should be avoided in patients with hematologic malignancies due to rare lymphoma risk associated with TNF-α inhibitors, particularly hepatosplenic T-cell lymphoma (HSTCL), which has been reported postmarketing with a very aggressive and often fatal disease course, especially in adolescent and young adult males receiving concomitant azathioprine or 6-mercaptopurine [22]. The drug should also be used cautiously in severe congestive heart failure and can induce rare hepatitis, requiring case-by-case decision-making in patients with concurrent ICI hepatitis and colitis [22]. Regarding cancer-related outcomes, the impact of infliximab on tumor response remains uncertain and somewhat controversial. While some retrospective analyses suggested potential associations with worse cancer outcomes, these findings may be confounded by concurrent high-dose corticosteroid use [22]. More recent data, including a large retrospective study of 185 patients, found no significant difference in best tumor response before and after infliximab in melanoma patients, and response to infliximab was actually associated with decreased risk of death (*p* = 0.0383 in melanoma) and longer progression-free survival in genitourinary cancers [37]. Interestingly, in one multicenter study conducted by Alexander et al., cancer non-progression was significantly more common in patients with infliximab-resistant enterocolitis (64.4%) compared with infliximab-responsive enterocolitis (37.5%), suggesting that prolonged and severe inflammatory side effects may correlate with better cancer outcomes [38]. Different clinical and histopathological features have been identified as potential predictors of infliximab response in ICI-induced colitis. For instance, in multivariable analysis, infliximab-resistant enterocolitis was associated with the presence of rectal bleeding (OR 0.19, 95% CI 0.04–0.80) and the absence of colonic crypt abscesses on histology (OR 2.16, 95% CI 1.13–8.05), suggesting that both symptom severity and histopathological patterns may help identify patients less likely to respond [38] (Table 1). Based on these observations, patients who do not respond to or are not eligible for infliximab may require alternative strategies.

#### 3.3.2. Vedolizumab

Vedolizumab, a gut-selective monoclonal antibody targeting the α4β7 integrin, is recommended as a second-line therapy for steroid-refractory ICI-induced colitis, either as an alternative to infliximab or for patients who fail infliximab therapy [22,28]. By specifically binding to the α4β7 integrin on memory T-lymphocytes and blocking its interaction with mucosal addressin cell adhesion molecule-1 (MAdCAM-1), vedolizumab inhibits the migration of gut-homing T-lymphocytes into inflamed gastrointestinal tissue without affecting systemic immunity, a mechanism that distinguishes it from systemic immunosuppressants and makes it particularly attractive in the oncologic setting [4,28]. Indeed, it is considered a preferred agent for managing IBD in patients with active or prior malignancy, as it theoretically preserves antitumor immunity while controlling intestinal inflammation; studies have shown no increase in the risk of new or recurrent cancer in IBD patients with prior malignancy treated with vedolizumab compared to anti-TNF agents or no immunosuppression [39,40]. Consistent with IBD protocols, dosing is 300 mg intravenously at weeks 0, 2, and 6, with the full induction regimen recommended to maximize mucosal healing and prevent recurrence [22,28]. Importantly, receipt of ≥3 doses has been associated with less frequent colitis recurrence and improved overall survival, underscoring the value of completing the induction course [41]. Efficacy data are robust across multiple study designs. One of the largest retrospective case series demonstrated sustained clinical remission in 86% (24/28) of patients after a median of three infusions, with endoscopic remission achieved in 54% at 6 months [42]. Notably, patients who had never received infliximab were more likely to achieve clinical remission with vedolizumab than those previously treated with infliximab (95% vs. 67%), suggesting potential benefit from earlier use in treatment algorithms [28]. Furthermore, a meta-analysis confirmed vedolizumab effectiveness in 85% (95% CI 60–96) of patients with ICI-induced colitis [35]. In early case series, steroid-free remission was achieved in a median of 56 days from vedolizumab initiation, with normalized fecal calprotectin [4]. More recent comparative data from a two-center observational study of 184 patients found comparable remission rates between vedolizumab and infliximab (89% vs. 88%), though vedolizumab was associated with shorter steroid exposure (35 vs. 50 days, *p* < 0.001), fewer hospitalizations (16% vs. 28%, *p* = 0.005), and lower colitis recurrence rates [41]. However, vedolizumab may have a slightly longer time to clinical response (17.5 vs. 13 days) compared to infliximab, which should be considered in patients requiring rapid symptom control [41]. The safety profile of vedolizumab is favorable, with early case series reporting no vedolizumab-related side effects [4]. The gut-selective mechanism of action limits systemic immunosuppression, potentially reducing infection risk compared to systemic agents like infliximab [28]. Before initiating vedolizumab, infectious disease screening (HIV; hepatitis A, B, C) and tuberculosis testing should be performed, though treatment should not be delayed in urgent situations [22]. A key advantage of vedolizumab is its reduced potential influence on cancer treatment and tumor response: the gut-selective mechanism of action does not interfere with the efficacy of immune checkpoint inhibitor therapy at systemic sites, making it an especially attractive option for ICI-induced colitis [28]. In keeping with this selective mechanism, safety data from conventional IBD are reassuring, with no excessive risk of cancer or serious infection detected, and vaccination studies confirm that systemic immunization is preserved despite suppression of gut immune responses to orally delivered vaccines [28]. At present, vedolizumab has not been shown to have a substantial influence on antitumor responses more generally, though use of these agents is highly correlated with high-dose systemic glucocorticoids, creating substantial bias in retrospective analyses [22]. However, vedolizumab may theoretically interfere with ongoing antitumor responses in the GI mucosa in patients receiving immunotherapy for primary GI malignancies or for tumors with GI metastases, warranting consideration in treatment selection [22]. The steroid-sparing effect of vedolizumab is clinically significant, as prolonged high-dose corticosteroid exposure has been associated with reduced progression-free survival and overall survival; notably, higher number of selective immunosuppressive therapy doses (≥3) was associated with better overall survival, while more steroid exposure resulted in worse patient outcomes [28,41] (Table 1).

**Table 1:** Summary of the major studies on infliximab and vedolizumab for ICI-induced colitis included in this review. Abbreviations: RR: remission rate, CTLA-4: Cytotoxic T-Lymphocyte Antigen 4, PD-1: Programmed Cell Death 1, PDL-1: Programmed Cell Death Ligand 1. Levels of evidence assigned according to the Oxford Centre for Evidence-Based Medicine 2011 Levels of Evidence: Level 1, systematic review of randomized trials; Level 2, individual randomized trial or observational study with dramatic effect; Level 3, non-randomized controlled cohort or comparative follow-up study; Level 4, case series, single-arm cohort or historically controlled study; Level 5, mechanistic reasoning or bench research. Systematic reviews and meta-analyses of non-randomized studies are rated at the level of their constituent studies.

INFLIXIMAB Anti-TNFα Monoclonal Antibody						
Study	Year	Study Design	N Patients	Efficacy	Safety	Level of Evidence
Ibraheim et al. [35]	2020	Systematic review + meta-analysis of non-randomized studies	333	Overall RR: 81% (95% CI 73–87; *p* = 0.01) Anti-CTLA-4: 78% (95% CI 68–85; *p* = 0.003) Anti-PD-1/L1: 100% (6/6)	Safety reported in 171/333 patients 11 infective episodes 3 hypersensitivity reactions (1 during 2nd dose)	3
Dahl et al. [33]	2022	Retrospective single-arm cohort study	140	Complete response: 73% (*n* = 101) Partial response: 17% (*n* = 14) No response: 10% (*n* = 14)	22 infective episodes (14 C. difficile; 1 death) 14 thromboembolic events	4
Zou et al. [41]	2021	Two-center retrospective comparative cohort study	94	Overall clinical remission: 88% (*n* = 83)	23 immunosuppressant-associated infections (25%)	3
VEDOLIZUMAB anti-α4β7 integrin monoclonal antibody						
Study	Year	Study design	N patients	Efficacy	Safety	Level of evidence
Abu-Sbeih et al. [42]	2018	Multicenter retrospective case series	28	Overall clinical remission: 86% (*n* = 24) Anti-CTLA-4: 25% (*n* = 6) Anti-PD-1/L1: 46% (*n* = 11) Combination therapy: 29% (*n* = 7)	Safety data not available	4
Ibraheim et al. [35]	2020	Systematic review + meta-analysis of non-randomized studies	50	Overall clinical remission: 85% (95% CI 60–96)	1 skin rash episode 1 diffuse joint pain	3
Zou et al. [42]	2021	Two-center retrospective comparative cohort study	62	Overall clinical remission: 89% (*n* = 55)	12 immunosuppressant-associated infections (19%)	3

For patients with ICI-induced colitis refractory to both infliximab and vedolizumab, several emerging therapies have been reported in case series and small studies, though evidence remains limited and mechanisms of action in this specific context are inadequately elucidated. These agents, including ustekinumab, tofacitinib, tocilizumab, adalimumab, golimumab, and abatacept, are considered in life-threatening cases when conventional biologics have failed [22,28].

Direct comparative data between infliximab and vedolizumab in ICI-induced colitis remain limited to retrospective cohorts, but consistent patterns have emerged across studies. Regarding speed of clinical response, infliximab seems to act faster: in the two-center study by Zou et al., median time to clinical response was 13 days with infliximab versus 17.5 days with vedolizumab (*p* = 0.012) [41]. A subsequent systematic review and meta-analysis pooling comparative cohorts confirmed a shorter median time to response with infliximab (13 versus 18 days, *p* = 0.012) [43]. Regarding recurrence, which may serve as a surrogate for durable mucosal healing, vedolizumab seems to perform better: a systematic review and meta-analysis of six retrospective cohorts (645 patients) found vedolizumab associated with significantly lower colitis recurrence than infliximab (OR 0.29, 95% CI 0.15–0.54) and shorter systemic steroid exposure (mean difference −16.88 days, 95% CI −20.47 to −13.30), with no significant difference in remission rates between the two monotherapies (OR 3.16, 95% CI 0.29–34.01) [43]. Regarding infectious risk, infliximab carries a higher burden of systemic immunosuppression: rates of infection-related hospitalization up to 24% and secondary gastrointestinal infections (predominantly C.difficile) in 16% of patients have been reported with infliximab [33], whereas vedolizumab’s gut-restricted mechanism is associated with a more favorable infectious profile and no excess risk of serious infection in comparative IBD safety data [28]. This distinction is particularly relevant in oncologic patients, who are frequently receiving concurrent chemotherapy or are otherwise immunocompromised. Finally, regarding preservation of anti-tumor immunity, the two agents differ mechanistically rather than empirically: infliximab neutralizes TNF-α systemically; instead, vedolizumab’s gut-selective actions should in principle spare systemic anti-tumor T-cell trafficking [4,28]. However, this theoretical advantage has not been confirmed by superior oncologic outcomes in comparative cohorts; the two-center study by Zou et al. found no significant difference in overall survival between agents when accounting for the number of biologic doses received [41].

#### 3.3.3. Ustekinumab

Ustekinumab, a monoclonal antibody targeting the p40 subunit shared by IL-12 and IL-23, has shown promising efficacy in refractory ICI-induced colitis. In the largest two-center experience, 68.4% (13/19) of patients achieved clinical remission with ustekinumab after failing steroids plus infliximab (57.9%) and/or vedolizumab (94.7%), with mean fecal calprotectin levels dropping significantly (629 to 92 mcg/mg, *p* = 0.0004) [44]. The rationale for IL-12/23 blockade is supported by mechanistic studies demonstrating that ICI-colitis is dependent on an IL-23/IFNγ axis, with pathogenic IFNG + IL17+ CD4+ T cells driven by IL-23 signaling from intestinal macrophages; in murine models, IL-23 blockade reduced pathogenic T cells and mitigated colitis severity [45,46].

#### 3.3.4. Tofacitinib

Tofacitinib, an oral JAK inhibitor, offers the advantage of rapid onset of action and has demonstrated efficacy in multi-refractory cases. In a multicenter observational study of 53 patients with various irAEs treated with tofacitinib, clinical remission rates were 96.7% in steroid-resistant cases and 100% in patients with steroid taper failure, with a favorable safety profile (7.5% infectious events) and median overall survival of 16.1 months from ICI initiation [47]. Case reports describe complete responses to tofacitinib in patients with life-threatening colitis refractory to corticosteroids, infliximab, and vedolizumab, with ongoing tumor response and no evidence of disease despite multiple immunosuppressive treatments [48,49]. Dosing is typically 10 mg twice daily for induction (adapted from ulcerative colitis protocols), with potential dose reduction to 5 mg twice daily for maintenance [50,51]. Safety considerations include venous thromboembolism and herpes zoster infection, warranting appropriate prophylaxis [50,52]. Notably, cancer itself is an independent and well-established risk factor for venous thromboembolism, with a four- to sevenfold increased risk compared to the general population [53]. Therefore, the use of tofacitinib in oncologic patients may represent a compounding thrombotic risk that warrants heightened vigilance and consideration of thromboprophylaxis. While no specific guidelines currently address venous thromboembolism prophylaxis in the setting of ICI-induced colitis, established recommendations exist for IBD patients: the ECCO guidelines on Extraintestinal Manifestations in IBD recommend the use of a prophylactic dose low-molecular-weight heparin or fondaparinux in the case of acute medical illness requiring hospitalization or after surgical interventions [54]. Moreover, in the same patients, in the case of acute thromboembolic events, use of direct oral anticoagulants (DOACs) at full therapeutic dosing is advised [54].

Regarding herpes zoster, the best preventive strategy consists in the administration of recombinant herpes zoster vaccine in every IBD patient or, alternatively, the administration of live zoster vaccine only in patients aged ≥50 years, according to the 2021 ECCO guidelines [55].

#### 3.3.5. Tocilizumab

Tocilizumab, an anti-IL-6 receptor monoclonal antibody, has shown efficacy in ICI-induced colitis and arthritis. In an open-label clinical study (COLAR), 79% (15/19) of patients achieved the primary endpoint of ≥1 grade CTCAE reduction within 8 weeks, with complete remission in 10 patients at week 24 without glucocorticoids [56]. In this trial, tocilizumab was administered intravenously at a dosage of 8 mg/kg every 4 weeks; the drug was well-tolerated with manageable adverse events, though caution is warranted in patients with clinically active diverticular disease due to increased risk of GI perforation [56].

#### 3.3.6. Other Therapies

Alternative anti-TNFα agents such as adalimumab and golimumab may have a role in patients developing infusion reactions to infliximab or preferring subcutaneous administration, though they are unlikely to be helpful as third-line agents for patients unresponsive to infliximab [28]. Successful use of adalimumab has been reported in steroid-refractory cases, sometimes with concomitant methotrexate [28].

Abatacept (CTLA-4-Ig) represents a mechanistically intriguing option, as it binds CD80 and CD86 to antigen-presenting cells, preventing their engagement with CD28 and thereby restoring the co-stimulatory brake that anti-CTLA-4 therapy is designed to release [22]. Its mechanistic rationale is consequently strongest for colitis arising after ipilimumab-containing regimens, which are also associated with the highest incidence and severity of gastrointestinal toxicity [21] and correspondingly weaker where CTLA-4 blockade was not the inciting agent [22]. Reported experience with abatacept in irAEs nonetheless derives almost entirely from organ systems other than the gastrointestinal tract; for ICI-induced colitis specifically, no cohort study or case series has evaluated the agent, so its inclusion among therapeutic options rests on mechanistic reasoning rather than on demonstrated intestinal efficacy [28]. This distinction matters, because the same property that makes the agent attractive raises the possibility that reversing the CTLA-4 blockade will abrogate the antitumor response together with the toxicity, a risk that the American Gastroenterological Association regards as substantial for abatacept as for ustekinumab and tofacitinib. This risk cannot presently be quantified, since no data exist on oncologic outcomes, treatment duration, or subsequent ICI rechallenge after its use in this setting [22]. Therefore, these therapies should be reserved for life-threatening cases [22].

As it stands, the impact of these emerging therapies on tumor progression remains a critical concern. Importantly, shorter durations of steroid treatment for colitis may be associated with less cancer progression, and the use of selective immunosuppressive therapy was not found to negatively impact progression-free survival in retrospective analyses [57]. Primary mechanistic work provides some reassurance in this regard. Single-cell analyses have shown that ICI-induced colitis is driven by macrophage-derived IL-23 acting on pathogenic IFNG+ IL17+ CD4+ T cells and that IL-23 blockade reduces these populations and attenuates colitis severity in murine models [45,46]. Importantly, these pathogenic T-cell populations have not been found to correlate with antitumor responses in patients responding to immunotherapy for colorectal cancer, raising the possibility that they can be targeted without compromising ICI efficacy [45,46]. This inference remains indirect, however, and has not been tested prospectively in patients receiving IL-23–directed therapy for colitis [45,46]. Nevertheless, given the limited data and theoretical concerns about systemic immunosuppression, these agents should be used judiciously with careful monitoring of both colitis response and tumor status.

The evidentiary basis for ustekinumab, tofacitinib, and other third-line agents in ICI-induced colitis remains substantially weaker than for infliximab and vedolizumab. The largest ustekinumab experience comprises 19 patients from a two-center series [44]; tofacitinib data derive mainly from a 53-patient multicenter observational study of mixed irAEs (not colitis-specific) [47], supplemented by isolated case reports [48,49], whereas no clinical series has evaluated abatacept in this indication, and its inclusion rests on mechanistic rationale alone [22]. None of these agents has been evaluated in a comparative or randomized design, and reported response rates (e.g., 68.4% for ustekinumab [44], 96.7–100% for tofacitinib in steroid-resistant or taper-failure irAEs [47]) come from heterogeneous, non-comparative cohorts. Isolated case reports add further texture to this picture, illustrating a genuine potential role for these agents in the small subset of patients who fail both infliximab and vedolizumab: tofacitinib-induced remission within days in a patient refractory to corticosteroids, infliximab and vedolizumab [58], and ustekinumab achieved clinical response in a patient who had additionally failed microbiota transplantation, representing a fourth line of therapy [59]. This evidence is valuable for signaling where these agents may fit in a refractory-disease scenario, but it remains difficult to estimate the true response rate in unselected refractory patients from the literature as it stands. This evidentiary gap directly influences their positioning: because ustekinumab, tofacitinib, and abatacept act via broader or less gut-restricted mechanisms than vedolizumab, there is a substantial theoretical risk of interference with antitumor immunity; therefore, they should be reserved for life-threatening, double-refractory disease rather than being used as routine third-line options [22]. Given this asymmetry between plausible efficacy and unresolved oncologic risk, use of these agents should be restricted to specialized centers with multidisciplinary oncology-gastroenterology input, with prospective registry data collection to build the evidence base that is currently missing.

### 3.4. Oncological Outcomes

A central premise of advanced therapy for ICI-induced colitis is that gastrointestinal toxicity can be controlled without compromising the antitumor effect of the ICI [22]. The available oncologic evidence, while still largely retrospective, is reassuring but incomplete [60]. When corticosteroid exposure is accounted for as a confounder, none of the advanced therapies have been consistently linked to worse survival outcome [41]. In the two-center cohort of 184 patients treated with infliximab or vedolizumab, receiving ≥3 doses of selective immunosuppressive therapy was independently associated with more favorable overall survival, while greater steroid exposure was associated with worse outcomes [41]. Similarly, a large retrospective study of 185 melanoma patients found no significant difference in best tumor response before and after infliximab, and infliximab response was associated with decreased risk of death (*p* = 0.0383) [37]. For vedolizumab, safety data extrapolated from IBD patients with prior malignancy show no increase in new or recurrent cancer compared with anti-TNF agents or no immunosuppression [39,40], though this evidence is indirect and not specific to the ICI-colitis population.

Regarding ICI rechallenge, the decision to resume immunotherapy after colitis resolution is common in clinical practice. In a pharmacovigilance analysis of 24079 irAEs, recurrence of the same event occurred in approximately 29% of rechallenged patients [61]; this estimate is pooled across all irAEs and is not specific to colitis. Concurrent maintenance biologic therapy on resumption appears to reduce recurrence without compromising survival [62]. In a three-center study of 138 patients who required infliximab or vedolizumab for initial colitis control, patients restarted on ICI with concurrent selective immunosuppressive therapy had a significantly lower rate of severe recurrence than those restarted on ICI alone (20.8% vs. 34.4.%; multivariate OR 0.34, *p* = 0.034), with no difference in overall survival or cancer progression rates between groups [62]. More broadly, across cancer types, ICI rechallenge after any irAE-related discontinuation has been associated with improved progression-free survival (HR 0.56, 95% CI 0.43–0.73) and overall survival (HR 0.55, 95% CI 0.43–0.72) compared with permanent discontinuation, in a meta-analysis of 36 studies and 2026 patients, although this analysis was not restricted to colitis, and rechallenge after disease progression (rather than after an adverse event) showed a less favorable safety profile [63]. Despite these encouraging signals, critical knowledge gaps persist. First, no prospective trial has been designed with dual oncologic and gastrointestinal endpoints; all survival data are derived from retrospective cohorts with inherent confounding by indication and by concurrent corticosteroid exposure [41,60]. Moreover, biomarkers capable of identifying patients in whom advanced therapy might blunt the antitumor immune response, as opposed to patients in whom it is oncologically neutral, have not been validated yet [22]. Addressing these gaps will require prospective, adequately powered trials incorporating tumor response, progression-free survival, and overall survival as co-primary endpoints alongside colitis-specific outcomes [60].

## 4. Discussion

As the clinical utility of immune-checkpoint inhibitors expands across a growing number of oncologic indications, the management of immune-related adverse events has become a primary focus of patient care. Colitis represents one of the most frequent and clinically significant toxicities, often leading to the permanent discontinuation of life-saving immunotherapy. While international guidelines establish corticosteroids as the first-line standard of care, a substantial proportion of patients fail to achieve a durable response. This high rate of steroid-refractoriness remains a critical unmet need, as delays in treatment escalation are associated with increased risks of bowel perforation, prolonged hospitalization, and poorer overall survival. To mitigate these risks and optimize the therapeutic window, a more proactive and evidence-based management strategy is required. The management of ICI-induced colitis hinges on two critical interventions: early endoscopic evaluation and timely biologic therapy. These elements are essential because the condition is characterized by rapid clinical changes, with symptoms often escalating over just a few days, particularly in ipilimumab-containing regimens, and a clinical presentation more reminiscent of colonic infection than IBD [22]. Delayed intervention can lead to life-threatening complications, including colonic perforation, toxic megacolon, and death (in approximately 2% of cases), underscoring the need for prompt action [23,64]. Retrospective analyses show that early endoscopy correlates with improved outcomes, while endoscopic identification of colonic ulceration is the primary established predictor of treatment response in ICI enterocolitis [22].

A further consideration is that ICI-induced colitis may behave as a delayed toxicity, with a variable and sometimes deferred onset [65]. Anti-CTLA-4-related colitis tends to emerge early, around 6–7 weeks, whereas anti-PD-1/PD-L1 colitis is less predictable and may appear at a median of roughly 25 weeks, occasionally up to two years after initiation [65,66]. Events occurring at least 90 days after ICI withdrawal have been termed delayed immune-related events (DIRE) [67]. The clinical significance of this phenomenon is increasing, since patients treated with curative intent receive a limited course of immunotherapy before entering surveillance, and reduced post-treatment vigilance may favor diagnostic misattribution and delayed management [67]. The resulting diagnostic hazard is twofold: delayed colitis may be misattributed to infection, to chemotherapy-related or radiation-related entheropathy, to postoperative functional change, or to disease progression, and patients no longer receiving active treatment may present to primary or emergency care rather than their oncology team, where the pharmacological history is less likely to be elicited [67]. A history of previous ICI exposure should therefore be sought in any patient presenting with new onset diarrhea, irrespective of the interval since the last dose, and the absence of current treatment should not lower diagnostic suspicion [67]. The diagnostic pathway for suspected DIRE colitis should be identical to that for on-treatment colitis, with early exclusion of infection and early endoscopy with biopsy, since the histological features that distinguish ICI-induced colitis from other causes do not depend on the temporal relationship to therapy [28,67]. Patients completing immunotherapy should receive counseling regarding persistent risk, and documentation of ICI exposure in a form accessible to non-oncological clinicians is a simple and probably underused safeguard [67]. No evidence base presently defines the optimal duration of surveillance or whether management of DIREs should differ from that of on-treatment colitis, and these questions will become more pressing as perioperative immunotherapy is adopted widely [68].

Emerging evidence further supports early biologic administration, which yields superior outcomes. For patients with severe colitis and high-risk endoscopic features such as ulcerations, early introduction of infliximab or vedolizumab can be considered to reduce recurrence and limit prolonged corticosteroid exposure [57]. In one retrospective study, early infliximab addition shortened median time to diarrhea resolution to 3 days (versus 9 days with corticosteroids alone), even in cases with higher-grade colitis [36]. Biologic responses are typically rapid (generally within 7 days, in contrast to IBD), and receipt of at least 3 doses is associated with lower colitis recurrence rates, favoring completion of the full induction regimen [22,41].

Despite the high efficacy and rapid response rates associated with primary biologic induction, a significant clinical challenge persists for the subset of patients who exhibit primary non-response or subsequent relapse. Since TNF-α inhibitors and α4β7 integrin antagonists operate through distinct pathways, therapeutic failure with one mechanism does not necessarily imply a global resistance to all biologics [18].

In this regard, current evidence suggests that when one immunobiological agent fails, a shift to a different class agent is reasonable. Patients who do not respond to the initial choice of biologic therapy should switch treatment class from infliximab to vedolizumab or vice versa [22]. Given the severity of these cases, waiting for a standard washout period is not recommended, and patients should be treated within a few weeks of their last infusion of the prior medication as soon as it is evident that they are not responding [22]. Should both vedolizumab and infliximab fail to achieve remission, salvage options like ustekinumab, tofacitinib, or abatacept may be explored, although the theoretical impact of these agents on oncologic outcomes warrants further investigation [22] (Figure 2).

Once clinical remission of the colitis is achieved, the priority often returns to the patient’s underlying malignancy and the potential resumption of immune checkpoint inhibitors. Given that these therapies are of fundamental importance for achieving durable survival in oncologic patients, the decision to re-challenge must carefully weigh the imperative for cancer control against the high risk of recurrent immune-mediated toxicity [23].

For grade IV (life-threatening) colitis, permanent discontinuation of the immunotherapy agent responsible for toxicity is recommended [23]. This includes cases with hemodynamic instability and serious complications such as ischemic bowel, perforation, or toxic megacolon [23].

For grade III colitis, guidelines commonly recommend suspending ICIs and the administration of high-dose corticosteroids, individualizing rechallenge based on tumor response, toxicity severity, alternatives, and recurrence risk (lower switching anti-CTLA-4 to anti-PD-1/PD-L1) [22,23,69,70]. However, some recommendations diverge across guidelines: for instance, NCCN allows anti-PD-1/PD-L1 monotherapy resumption post-resolution with concurrent advanced therapy prophylaxis [69]; ASCO/AGA supports retreatment if there are no other effective cancer treatments available [22,23]; and European Delphi Consensus emphasizes early multidisciplinary management and the use of biologics for high-risk endoscopic findings [70] (Table 2).

While guidelines diverge on rechallenge strategy, they show notably stronger consensus on first-line biologic selection: a 2024 systematic review comparing different guidelines’ recommendations across irAEs found the guidelines concordant in recommending infliximab as first-line biologic therapy for corticosteroid-resistant colitis [70]. This concordance is specific to colitis: the same guidelines diverge on infliximab use in other irAEs, such as hepatitis and myocarditis [70].

For grade II diarrhea/colitis, ICI may be resumed once symptoms have resolved to grade I or less [23]. In rare circumstances where the patient cannot completely taper off steroids, immunotherapy may be resumed while the patient is still on less than 10 mg prednisone equivalent daily [23]. Concurrent advanced therapy on immunotherapy resumption should be considered, as maintenance immunosuppressive therapy has been associated with significantly lower colitis recurrence (17% vs. 37%, *p* = 0.027) and more extended ICI treatment while preserving similar overall survival [71]. To document mucosal healing, before resumption of ICI therapy, it is suggested to repeat a colonoscopy; moreover, serial monitoring of fecal calprotectin levels while on treatment (every 2 months) may be helpful to guide treatment duration until achieving endoscopic remission [22,41] (Table 3).

Importantly, the development of colitis with one ICI does not necessarily prohibit the use of another. Patients who switch ICI classes still have risk for recurrent irAEs but generally have lower risk if they switch from ipilimumab to PD-1/PD-L1 blocking therapy [22]. In a multicenter retrospective study, the risk of recurrent ICI-induced colitis was approximately 30% for most regimens but appeared to be higher in patients who developed enterocolitis on PD-1/PD-L1 inhibitors and then switched to CTLA-4 inhibitors [22]. Even in the setting of recurrent ICI-induced colitis, standard therapies typically remain effective, and enterocolitis before ICI initiation should not be considered an absolute contraindication for future immunotherapy if ICIs are the only available options [22].

While the management of irAEs presents clinical challenges, their occurrence may paradoxically carry prognostic significance. Multiple meta-analyses have demonstrated that irAEs are associated with enhanced tumor response and increased overall survival. Pooled data from 52 studies comprising 9156 patients demonstrated a statistically significant greater probability of achieving objective tumor response for patients with irAEs compared to those without (OR 3.91, 95% CI 3.05–5.02), with prolonged progression-free survival (PFS) (HR 0.54, 95% CI 0.46–0.62) and overall survival (OS) (HR 0.51, 95% CI 0.41–0.59) [72]. This association is particularly robust for skin and endocrine irAEs, while high-grade toxicities (G3–5) were not associated with significantly favorable PFS or OS [72,73]. Notably, low-grade irAEs (grades 1–2) are linked to better survival, unlike high-grade events [73,74].

Beyond current therapeutic strategies (Table 4), the management of ICI-induced colitis necessitates a multidisciplinary approach, integrating the expertise of gastroenterologists, oncologists, surgeons, and pathologists to balance the dual imperatives of controlling gastrointestinal toxicity and preserving oncological outcomes [70]. Current therapeutic algorithms are largely extrapolated from IBD protocols, yet a considerable proportion of agents effective in IBD, including selective IL-23 inhibitors such as risankizumab, guselkumab, and mirikizumab, remain untested in the ICI-induced colitis population. Furthermore, the role of intestinal ultrasound as a rapid, non-invasive, and radiation-free monitoring tool warrants investigation, particularly given its proven utility in IBD surveillance [75]. A critical limitation in the field is the absence of standardized protocols for endoscopic and histological assessment; dedicated scoring systems for ICI-induced colitis do not exist, and the applicability of established IBD indices remains undefined. Addressing these gaps through prospective validation studies will be essential to refine disease monitoring and optimize therapeutic decision-making in this complex patient population.

**Table 2:** Comparison of major international guideline recommendations for the management of ICI-induced colitis by severity grade and stance on immune checkpoint inhibitor rechallenge. Abbreviations: ASCO: American Society of Clinical Oncology; AGA: American Gastroenterological Association; ESMO: European Society for Medical Oncology; BSG British Society of Gastroenterology; NCCN: National Comprehensive Cancer Network; ICI: immune checkpoint inhibitor.

Guideline (Year)	Grade 2 Colitis	Grade 3–4 Colitis	ICI Rechallenge After Resolution
ASCO (2021) [23]	Oral corticosteroids; consider withholding ICI	Hold ICI; IV corticosteroids; infliximab/vedolizumab if no response in 3–5 days	Individualized; supports treatment if no other effective cancer treatment available
AGA Clinical Practice Update (2021) [22]	Corticosteroids; consider early biologic in high-risk endoscopic features	Infliximab or vedolizumab; switch class on primary non-response without full washout	Endoscopic/biomarker guided; repeat colonoscopy or serial fecal calprotectin before resumption
ESMO (2022) [76]	Oral corticosteroids; vedolizumab or infliximab for non-responders	High-dose IV corticosteroids; infliximab if no response in 3–5 days	Individualized on grade and multidisciplinary discussion
BSG (2020) [28]	Corticosteroids; biologic escalation per severity	Infliximab or vedolizumab; salvage agents (ustekinumab, tofacitinib) for double-refractory disease	Deferred to oncology team
NCCN (2020) [69]	Corticosteroids	Infliximab or vedolizumab	Allows anti-PD-1/PD-L1 monotherapy resumption post-resolution with concurrent advanced-therapy prophylaxis
European Delphi Consensus (2023) [68]	Early multidisciplinary evaluation	Biologics prioritized for high-risk endoscopic findings	Emphasizes early multidisciplinary management over fixed algorithm

**Table 3:** Comparison between ICI-induced colitis and IBD. Abbreviations: ICI: immune-checkpoint inhibitor, CTCAE: Common Terminology Criteria for Adverse Events, UC: ulcerative colitis, CD: Crohn’s disease, MRI: magnetic resonance imaging, CT: computed tomography, IUS: intestinal ultrasound, CRP: C-reactive protein, ESR: erythrocyte sedimentation rate.

	ICI-Induced Colitis	Inflammatory Bowel Disease (UC and CD)
▸ Symptoms	▸ Acute onset Diarrhea (hallmark), ± blood/mucus Abdominal pain, cramping, urgency Onset around 6 weeks after ICI start CTCAE grading (G1–G5)	▸ Chronic/relapsing Diarrhea, rectal bleeding, weight loss Abdominal pain, fatigue, malnutrition Extraintestinal manifestations Mayo (UC)/Harvey–Bradshaw (CD) scores
▸ Diagnosis	Endoscopy + biopsy (reference standard) CT scan if complications suspected	Endoscopy + biopsy (reference standard) MRI/CT enterography (CD) IUS
▸ Endoscopic appearance	Left-sided predominance (95%) Erythema, edema, loss of vascular pattern Ulcerations (high-risk feature) Resembles acute infectious colitis	UC: erythema, decreased vascular pattern, mucosal friability CD: skip lesions, cobblestoning, deep ulcers, fistulas, strictures
▸ Histological appearance	CD8+ dominant (CD8/CD4 > 1.17) Less prominent basal plasmocytosis (14%) Less frequent crypt distortion (23%) Acute pattern	CD4+ dominant More prominent basal plasmocytosis (92%) More frequent crypt distortion (75%) Chronic active inflammation, granulomas (CD)
▸ Biomarkers	Fecal calprotectin/lactoferrin CD8/CD4 ratio on biopsy (>1.17) No validated serum biomarker	Fecal calprotectin CRP ESR
▸ Therapy	▸ Stepwise/ICI withholding G1: supportive; continue ICI G2–3: withhold ICI + corticosteroids Steroid-refractory: infliximab or vedolizumab G4: permanent ICI discontinuation	▸ Long-term maintenance Mild UC: 5-ASA Moderate–severe: steroids → biologics Anti-TNF, vedolizumab, ustekinumab, IL-23 inhibitors JAK inhibitors (tofacitinib, upadacitinib)

**Table 4:** Comparison of advanced therapies used in ICI-colitis. Levels of evidence follow the Oxford Centre for Evidence-Based Medicine 2011 scheme: Level 1, systematic review of randomized trials; Level 2, individual randomized trial; Level 3, non-randomized controlled cohort or comparative follow-up study; Level 4, case series or single-arm cohort; Level 5, mechanistic reasoning. Abbreviations: CTCAE, Common Terminology Criteria for Adverse Events; GI, gastrointestinal; ICI, immune checkpoint inhibitor; irAE, immune-related adverse event; IV, intravenous; LoE, level of evidence; OS, overall survival; VTE, venous thromboembolism.

Positioning	LoE	Oncological Data	Key Safety Signals	Durability	Time to Response	Efficacy	Dosing	Agent (Target)
First-line advanced therapy when rapid symptom control is the priority	3	No significant change in best tumor response before/after treatment (n = 185); response associated with reduced risk of death in melanoma (*p* = 0.0383) [37]	Infection-related hospitalization 24%; secondary GI infection 16%, mostly C. difficile; thromboembolic events 10% at 90 days [33].	Higher recurrence than vedolizumab (OR 0.29 favoring vedolizumab) [43]; ≥3 doses associated with lower recurrence [41]	Median symptom improvement 3 days; median clinical response 13 days [33,41]	Pooled response 81% (95% CI 73–87) [35]; complete response 73% in a 140-patient cohort [33]; clinical remission 88% [41]	5 mg/kg IV, weeks 0, 2, 6	Infliximab (anti-TNFα)
First-line advanced therapy when infection risk or steroid-sparing is the priority	3	Indirect (IBD with prior malignancy): no increase in new or recurrent cancer in IBD patients with prior malignancy [39,40], not specific to ICI setting; no OS difference versus infliximab after adjustment for dose number [41]	Favorable; gut-selective mechanism limits systemic immunosuppression; no excess serious infection in comparative IBD safety data [28]	Lower colitis recurrence than infliximab (OR 0.29) [43]; shorter steroid exposure (mean difference −16.88 days) [43]	Median clinical response 17.5 days [41]; steroid-free remission at a median of 56 days [4]	Clinical remission 86% (24/28) [42]; pooled effectiveness 85% (95% CI 60–96) [35]; clinical remission 89% [41]; endoscopic remission 54% at 6 months [42]	300 mg IV, weeks 0, 2, 6	Vedolizumab (anti-α4β7 integrin)
Salvage, double-refractory disease	4	No direct data; mechanistic data suggest targeted IFNG + IL17 + CD4+ T cells are not associated with antitumor immunity [45,46]	Not systematically reported	Not reported	Not reported	Clinical remission 68.4% (13/19) after failure of steroids plus infliximab and/or vedolizumab; mean fecal calprotectin 629 to 92 mcg/mg (*p* = 0.0004) [44]	Per IBD protocol	Ustekinumab (anti-IL-12/23 p40)
Salvage, double-refractory disease	4	Median OS 16.1 months from ICI initiation in the mixed-irAE cohort [47]; not colitis-specific	Infectious events 7.5% [47]; venous thromboembolism and herpes zoster, compounded by the 4 to 7-fold baseline VTE risk conferred by malignancy [50,52,53]	Not reported	Rapid; response within days in case reports [58]	Clinical remission 96.7% in steroid-resistant and 100% in steroid-taper-failure cases, in a 53-patient mixed-irAE cohort [47]; case reports in triple-refractory colitis [48,49,58]	10 mg twice daily induction; 5 mg twice daily maintenance	Tofacitinib (JAK inhibitor)
Salvage; the only agent evaluated in a prospective open-label study	4	Not reported	Well tolerated; caution in clinically active diverticular disease owing to GI perforation risk [56]	Complete remission sustained to week 24 in 10/19 [56]	Within 8 weeks (primary endpoint window) [56]	≥1 CTCAE grade reduction within 8 weeks in 79% (15/19); complete remission without glucocorticoids in 10 patients at week 24 [56]	8 mg/kg IV every 4 weeks	Tocilizumab (anti-IL-6 receptor)
Alternative to infliximab for infusion reactions or subcutaneous preference; unlikely to help after infliximab failure	4	Not reported	Class effects as for infliximab	Not reported	Not reported	Successful use reported in steroid-refractory cases, occasionally with methotrexate [28]	Per IBD protocol, subcutaneous	Adalimumab / golimumab (anti-TNFα)
Last resort, life-threatening disease, specialist centers	5	Greatest theoretical risk of interfering with antitumor response; opposes anti-CTLA-4 blockade [22]	Infection risk; no ICI-colitis safety data	Not reported	Not reported	No clinical series in ICI-colitis; efficacy not established [22]	Per rheumatological protocol	Abatacept (CTLA-4-Ig)

## 5. Conclusions

ICI-induced colitis requires prompt recognition and individualized treatment. While corticosteroids remain first-line therapy, a substantial minority of patients require escalation to advanced therapy. Both infliximab and vedolizumab demonstrate high efficacy in steroid-refractory cases, with vedolizumab offering a steroid-sparing effect and infliximab providing faster symptom resolution. For multi-refractory patients, ustekinumab, tofacitinib, and tocilizumab offer additional options, though evidence regarding efficacy, safety and oncologic outcomes remains limited.

Current guidelines are based largely on retrospective data, and critical knowledge gaps persist regarding optimal biologic selection, risk stratification, and predictive biomarkers.

Randomized comparisons of first-line advanced therapies, powered for both gastrointestinal and oncological endpoints, are needed to measure rather than infer the effect of immunosuppression on antitumor activity. Prospectively validated biomarkers of corticosteroid refractoriness, whether mucosal, circulating, or microbial, would allow earlier escalation in patients at risk while sparing others. A disease-specific endoscopic and histological index would improve comparability across studies, and prospective registries represent the most realistic source of evidence for salvage agents, which the rarity of double-refractory disease places beyond the reach of randomized trials.

## Figures and Tables

**Figure 1:**
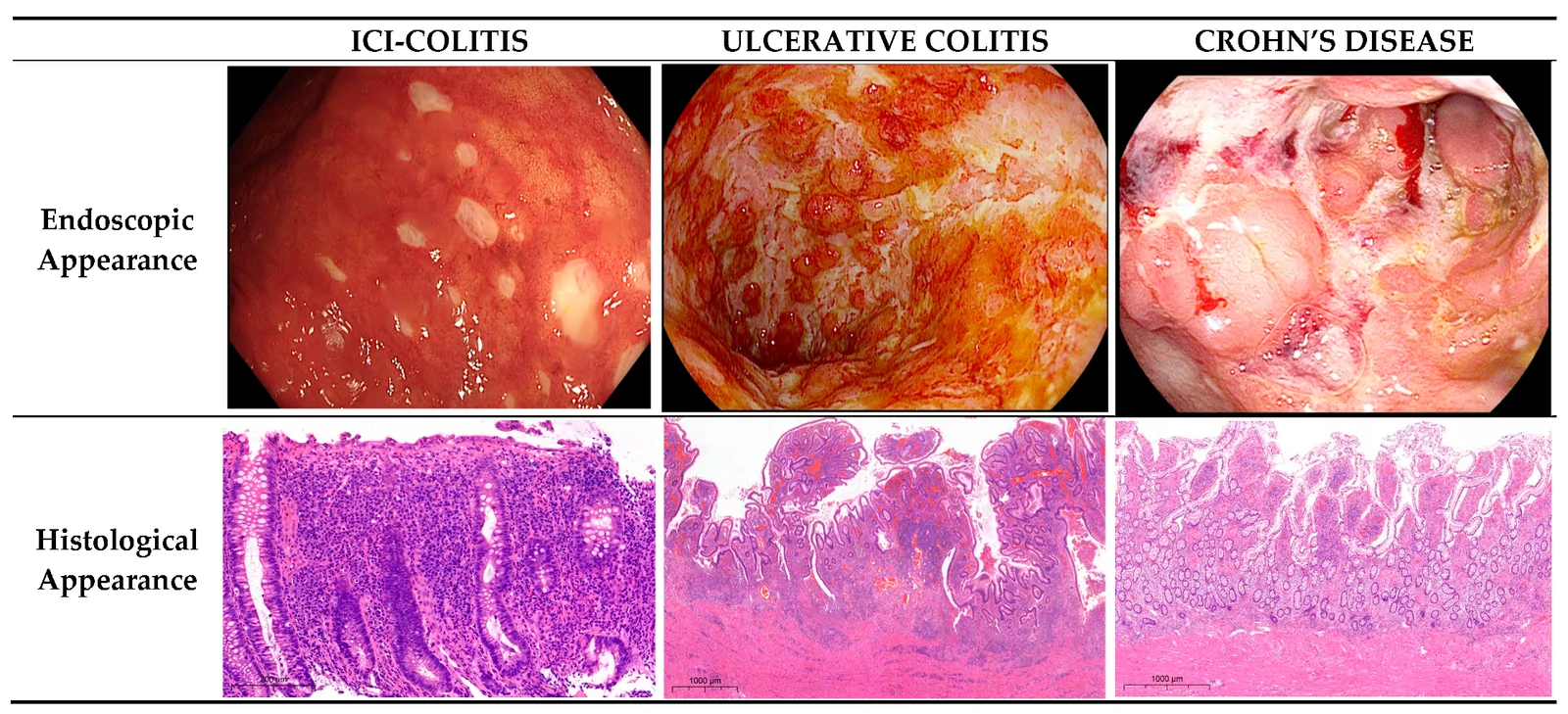
Endoscopic and histological comparison between ICI-colitis, UC, and CD. In ICI-colitis, the involvement of the mucosal and submucosal layer by lymphoid cells does not affect the glandular architecture, which is often preserved, with well-aligned and non-hypotrophic crypts; active inflammation of the lamina propria and epithelia is observed, and colocyte apoptosis can be seen; conversely, in IBD, the glandular architecture is altered by distortion and hypotrophy of the glandular adenomeres, the infiltrate presents a heteromorphic (CD) or predominantly plasma cell (UC) appearance with active inflammation variably extended to the lamina propria and epithelia, and a constant presence of plasma cells in the basal part of the lamina propria (basal plasmacytosis).

**Figure 2:**
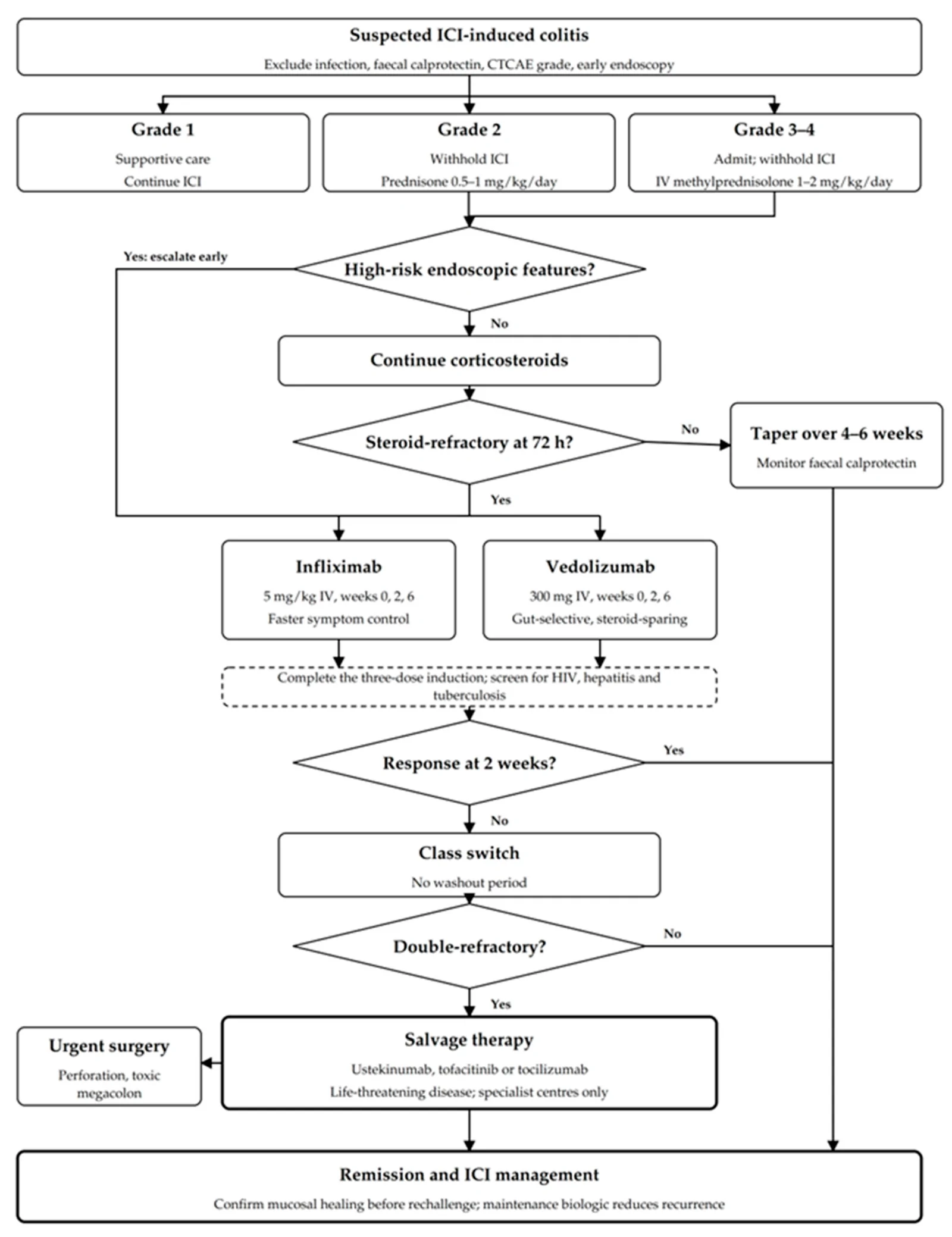
Stepwise algorithm for the management of immune checkpoint inhibitor-induced colitis. Decision points are based on CTCAE grade, endoscopic findings, steroid-refractoriness criteria, and prior biologic exposure. Salvage agents should be restricted to life-threatening, double-refractory disease managed in specialist centers. Abbreviations: CTCAE, Common Terminology Criteria for Adverse Events; ICI, immune checkpoint inhibitor; IV, intravenous.

## Data Availability

No new data were created or analyzed in this study. Data sharing is not applicable to this article.

## Supplementary Materials

The following supporting information can be downloaded at https://www.mdpi.com/article/10.3390/cancers18193108/s1: Table S1: Search strategy.
