# Supplementary material for: Advanced Therapies for the Management of Immune Checkpoint Inhibitor-Induced Colitis
Source: Cancers (Basel). 2026 Sep 24;18(19):3108. doi: 10.3390/cancers18193108 (PMC13664336; doi:10.3390/cancers18193108)
Supplement: Supplementary file 1 [file cancers-18-03108-s001.zip › cancers-4547429-supplementary.pdf]

Table S1. Search strategy.

Item	Detail
Design	Narrative, expert-led review; not registered; PRISMA not applicable
Databases	PubMed/MEDLINE; Embase; Scopus
Date range	Inception to 30th June 2026
Concept 1: condition	"immune checkpoint inhibitor" OR "immune checkpoint blockade" OR ipilimumab OR nivolumab OR pembrolizumab OR atezolizumab OR durvalumab OR "PD-1" OR "PD-L1" OR "CTLA-4"
Concept 2: toxicity	colitis OR enterocolitis OR diarrhea OR "immune-related adverse event" OR irAE OR "immune-mediated diarrhea"
Concept 3: comparator disease	"inflammatory bowel disease" OR "Crohn disease" OR "ulcerative colitis"
Concept 4: intervention	"advanced therapy" OR biologic* OR infliximab OR vedolizumab OR ustekinumab OR tofacitinib OR tocilizumab OR adalimumab OR golimumab OR abatacept OR "JAK inhibitor" OR corticosteroid*
MeSH terms (PubMed)	Immune Checkpoint Inhibitors; Colitis; Inflammatory Bowel Diseases; Biological Products; Janus Kinase Inhibitors
Emtree terms (Embase)	immune checkpoint inhibitor; colitis; inflammatory bowel disease; biological therapy
Combination	(Concept 1 AND Concept 2) AND (Concept 3 OR Concept 4)
Inclusion criteria	Adult patients; ICI-induced colitis or enterocolitis; report of treatment with corticosteroids, biologic agents or small molecules; original data, systematic review, meta-analysis, consensus statement or guideline
Exclusion criteria	Non-English; no extractable treatment or outcome data; paediatric-only populations; abstracts without accompanying peer-reviewed data; topic not pertinent
Screening	Independent title/abstract screening by two authors (C.S.M., F.F.); full-text assessment of potentially eligible records;
Supplementary searching	Hand-search of reference lists of included articles and relevant reviews
